# Acute kidney injury in an HIV patient with plasmablastic lymphoma – A double-edged sword

**DOI:** 10.4102/sajid.v39i1.637

**Published:** 2024-05-31

**Authors:** Gerhard van Wyk, Liezel Coetzee, Mogamat-Yazied Chothia

**Affiliations:** 1 Department of Nephrology, Faculty of Medicine and Health Sciences, Stellenbosch University, Cape Town, South Africa; 2 Department of Pathology, Faculty of Medicine and Health Sciences, Stellenbosch University, Cape Town, South Africa

**Keywords:** acute kidney injury, light chains, cast nephropathy, HIV, tumour infiltration

## Abstract

HIV patients frequently develop acute kidney injury (AKI) because of sepsis and diarrhoeal disease. Here, we report a case of an HIV-positive man with partially treated sinonasal plasmablastic lymphoma (PBL) and unexplained AKI. A kidney biopsy revealed two pathological processes.

**Contribution:**

While urinary tract obstruction is the most common mechanism by which PBL causes AKI, maintaining a high level of suspicion for multiple pathological processes in cases involving light chain producing PBL.

## Introduction

People living with HIV frequently develop acute kidney injury (AKI) because of sepsis and diarrhoeal disease.^1^ Another less common cause of AKI is lymphoma, which is frequently of B cell origin. It may affect the kidneys by several mechanisms that include lymphomatous infiltration of kidney parenchyma (LIK), urinary tract obstruction, tumour lysis syndrome or consequences of nephrotoxic chemotherapy.^2,3^ Consequently, it has a wide spectrum of clinical manifestations, which range from occult disease to various degrees of proteinuria and severe kidney failure.^3,4^

This case report presents a 43-year-old HIV-positive male who was known with partially treated right-sided sinonasal plasmablastic lymphoma (PBL) but got lost in the system and was not followed up. He returned 3 years later, this time with a left-sided sinonasal PBL and associated AKI. Further investigations revealed two pathological processes as the cause of AKI.

### Ethical considerations

An application for full ethical approval was made to the Health Research Ethics Committee (HREC) of Stellenbosch University and ethics consent was received on 16 February 2024. The ethics approval number is C23/12/031. Verbal informed consent was obtained from the patient involved in the case report.

## Case presentation

A 43-year-old man was diagnosed with HIV in 2017. His initial antiretroviral therapy regimen included tenofovir disoproxil fumarate (TDF), lamivudine (3TC), and efavirenz until 2020. Subsequently, he was transitioned to TDF, 3TC, and dolutegravir and maintained consistently undetectable viral loads for the past three years. He presented to his local hospital due to epistaxis that required urgent evaluation by an otorhinolaryngologist after conservative measures for bleeding control were ineffective. Upon evaluation, he was found to have a left sinonasal mass. Computed tomography of the sinuses revealed a poorly enhancing homogeneous soft tissue mass in the left maxillary antrum with extension of the mass superiorly into the ethmoid air cells, posteriorly into the left sphenoid sinus and nasopharynx, and into the left and right nasal passage as well as scalloping of the various bony elements making out the above-mentioned structures.

The patient who was diagnosed on a right-sided sinonasal mass biopsy 3 years prior to this presentation had a history of partial treatment for stage IV PBL. He was treated with two cycles of chemotherapy, initially with doxorubicin, cyclophosphamide, prednisone, and vincristine, followed by a second cycle of vincristine, methotrexate, and bleomycin; but the patient got lost in the system and was not followed up.

On the current admission, the patient had a blood pressure of 145/82 mmHg and a heart rate of 88 beats per minute. His clinical examination was notable for conjunctival pallor and hydration status with considered normal. There was no hepatosplenomegaly. The remainder of the clinical examination was unremarkable.

Routine blood tests revealed AKI and anaemia (Table 1). The HIV viral load was less than 20 copies per millilitre. Urinalysis was notable for nephrotic-range proteinuria and an inactive urine sediment was observed on microscopy. The serum protein electrophoresis revealed a monoclonal peak of 4 g/L and serum immunofixation demonstrated IgA lambda in the beta region and prominent free lambda in the gamma region (Table 1). A prominent lambda monoclonal band was also identified on urine protein electrophoresis and immunofixation. The kidney ultrasound revealed bilaterally enlarged echogenic kidneys (left 130.2 mm and right 130.0 mm) with reduced corticomedullary differentiation.

**TABLE 1:** Laboratory results.

Laboratory test (Reference range)	Results
Sodium (136 mmol/L – 145 mmol/L)	135.00
Potassium (3.5 mmol/L – 5.1 mmol/L)	5.40
Urea (2.1 mmol/L – 7.1 mmol/L)	27.80
Creatinine (64 µmol/L – 104 µmol/L)	1054.00
eGFR (> 90 mL/min per 1.73 m^2^)	5.00
Calcium (2.15 mmol/L – 2.50 mmol/L)	2.25
Magnesium (0.63 mmol/L – 1.05 mmol/L)	0.96
Phosphate (0.78 mmol/L – 1.42 mmol/L)	2.57
Uric acid (0.21 mmol/L – 0.43 mmol/L)	0.98
Total protein (60 g/L – 78 g/L)	67.00
Serum albumin (35 g/L – 52 g/L)	30.00
Total bilirubin (5 µmol/L – 21 µmol/L)	3.00
Alanine transaminase (10 U/L – 40 U/L)	24.00
Aspartate transaminase (15 U/L – 40 U/L)	63.00
Alkaline phosphatase (53 U/L – 128 U/L)	173.00
Gamma-glutamyl transferase (< 68 U/L)	66.00
Lactate dehydrogenase (100 U/L – 190 U/L)	869.00
Leucocyte count (3.92–10.4 × 10^9^/L)	8.18
**Differential cell counts**	
Neutrophil (1.6–6.98 × 10^9^/L)	4.27
Lymphocyte (1.4–4.2 × 10^9^/L)	2.53
Monocyte (0.30–0.80 × 10^9^/L)	1.21
Eosinophil (0.00–0.95 × 10^9^/L)	0.14
Basophil (0.00–0.10 × 10^9^/L)	0.03
Haemoglobin (13–17 g/dL)	7.60
Mean cell volume (83.1 fL – 101.6 fL)	88.20
Platelet count (171–388 × 10^9^/L)	323.00
Total cholesterol (< 5.0 mmol/L)	3.25
HIV viral load (copies/mL)	< 20.00
Anti-nuclear antibody	Positive at 1:40
PR3-ANCA	Negative
MPO-ANCA	Negative
Treponema pallidum antibody	Negative
Hepatitis B surface antigen	Negative
Hepatitis B surface antibody	Positive
Hepatitis C total antibody	Negative
**Serum protein electrophoresis**	
Alpha-1 globulin (2 g/L – 4 g/L)	4.00
Alpha-2 globulin (4 g/L – 8 g/L)	8.00
Total beta globulin (5 g/L – 10 g/L)	19.00
Beta-1 globulin	5.00
Beta-2 globulin	14.00
Gamma globulin (6 g/L – 12 g/L)	14.00
Paraprotein (0 g/L)	4.00
Serum free kappa light chains (3.3 mg/L – 19.4 mg/L)	159.60
Serum free lambda light chains (5.7 mg/L – 26.3 mg/L)	9433.00
Kappa-to-lambda ratio (0.36–3.1†)	0.02
Serum immunofixation	IgA lambda in beta-region with a prominent free lambda in the gamma region
**Urine**	
Urine protein electrophoresis and immunofixation	Prominent band: Monoclonal free lambda
Urine protein-to-creatinine ratio (< 0.15 g per day)	3.57

eGFR, estimated glomerular filtration rate; HIV, human immunodeficiency virus; ANCA, anti-neutrophilic cytoplasmic antibodies; PR3, proteinase 3; MPO, myeloperoxidase.

† , with kidney dysfunction.

A biopsy of the new left sinonasal mass confirmed a relapse of PBL. A bone marrow aspirate and trephine biopsy revealed hypercellular bone marrow with markedly increased atypical lymphocytes, the morphological and immunohistochemical features suggesting extensive involvement by PBL with a tumour burden of 90%.

**FIGURE 1:**
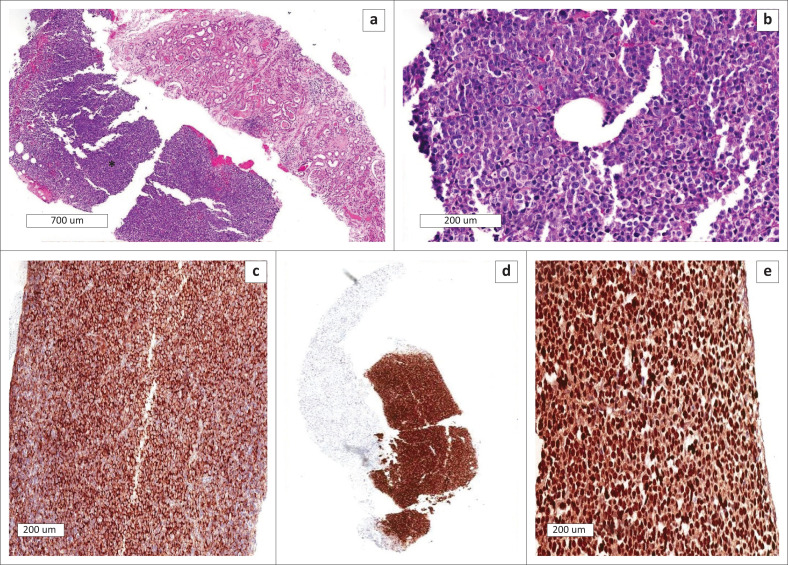
Histopathology of plasmablastic lymphoma kidney infiltration. (a) Low power haemotoxylin and eosin view of the plasmablastic lymphoma and kidney cortex (asterisk). (b) High power haemotoxylin and eosin view of large plasmablastic cells with frequent mitotic figures. (c) Diffuse CD138 membranous positive staining in the lymphoma. (d) Diffuse MUM1 nuclear positive staining in the lymphoma. (e) Diffuse nuclear positive Epstein–Barr encoding ribonucleic acid *in situ* hybridisation in the lymphoma.

**FIGURE 2:**
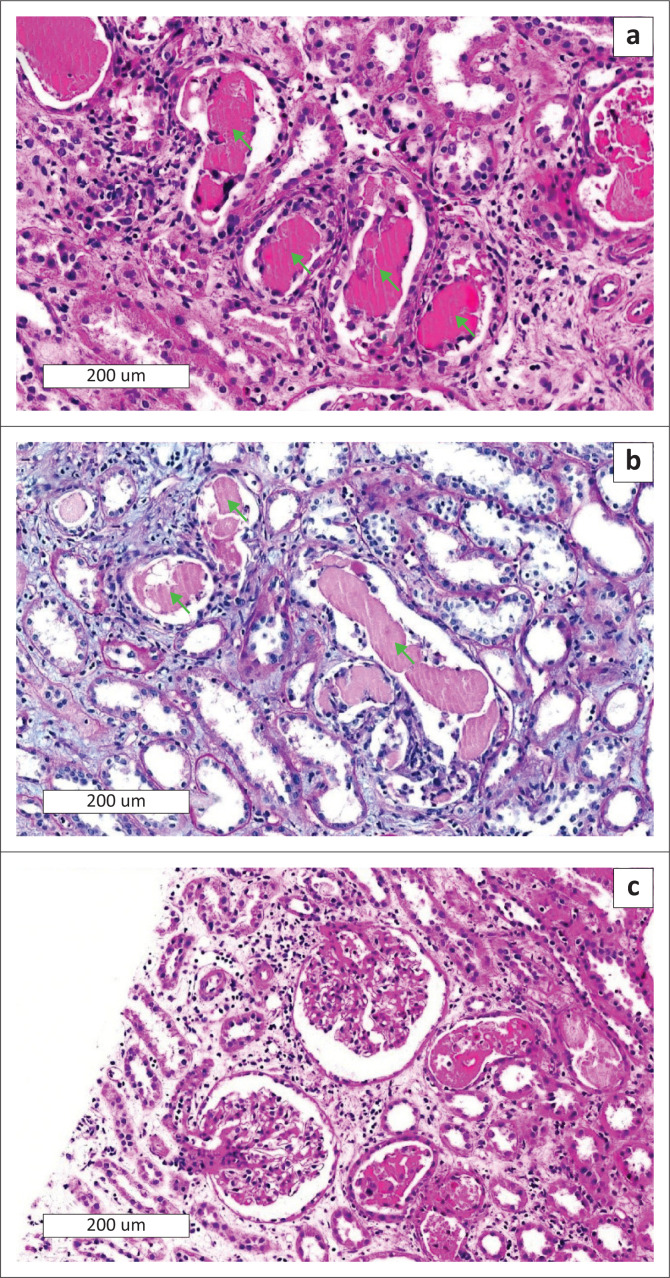
Histopathology of tubular casts. (a) High power view of the haemotoxylin and eosin section shows renal cortex with eosinophilic tubular casts and surrounding mononuclear cells (arrows). (b) The casts stain weakly positive with periodic acid Schiff (arrows). (c) Haemotoxylin and eosin section shows unremarkable glomeruli.

He was referred to nephrology to investigate the AKI in association with nephrotic-range proteinuria. An ultrasound-guided kidney biopsy was performed. Light microscopy of the kidney biopsy revealed infiltration by a haematolymphoid neoplasm with plasmablastic morphology, frequent apoptotic bodies, and high proliferation rate. The tumour cells stained diffusely positive with MUM1 and CD138 immunohistochemical stains as well as Epstein–Barr encoding ribonucleic acid (EBER) *in situ* hybridisation (Figure 1a–e). In addition, the tubulointerstitial compartment showed eosinophilic tubular casts with fracture planes, multinucleated giant cells, and acute tubular injury. An interstitial inflammatory reaction, predominantly with mononuclear inflammatory cells and few eosinophils accompanied the tubular casts. The glomeruli were normal in appearance and the vascular compartment showed hyaline arteriolosclerosis. The Congo red stain was negative for amyloidosis and the tubular casts were weakly periodic acid-Schiff stain positive (Figure 2a–c). Immunofluorescence staining for IgA, IgG, IgM and complement 3 were negative with a lambda-restricted light chain expression pattern in the tubular casts and PBL. Electron microscopy did not demonstrate any amyloid fibrils. There were no features suggestive of TDF-induced mitochondrial toxicity on electron microscopy. Findings were in keeping with PBL infiltration of the kidney parenchyma as well as light chain cast nephropathy (LCCN).

As a result of the extent of disease and severe kidney dysfunction, the patient received palliative care. His ART was changed to a non-nephrotoxic regimen, and he was discharged home with palliative care.

## Discussion

To the best of our knowledge, this is the first description of two pathological processes causing AKI in an HIV patient with light chain producing PBL, with both direct tumour infiltration of kidney tissue, as well as evidence of LCCN. Plasmablastic lymphoma is rare, accounting for only 2% of HIV-associated lymphomas.^5^ The World Health Organization has classified PBL as a subtype of diffuse large B-cell lymphoma and has a strong link with the Epstein–Barr virus infection with EBER detected in up to 80% of lymphomatous cells, as was observed in our case.^5^

Plasma cell dyscrasias may affect the kidneys by several mechanisms.^6^ These include glomerular diseases such as monoclonal light chain deposition disease, AL-amyloidosis and cryoglobulinaemia; while tubulointerstitial lesions include LCCN (most common), proximal tubular injury with resultant Fanconi syndrome and/or acute tubular necrosis, and direct tumour cell infiltration.^6^

Subclinical LIK is thought to be a common occurrence, the incidence reported to be between 6% and 60% of cases.^7^ In a large series of 696 cases, nearly a third of post-mortem examinations revealed LIK.^7^ As a result of its subtle and non-specific clinical manifestations, it is often occult and is diagnosed antemortem in only 14% of patients with lymphoma.^8,9^ Kidney biopsy findings typically include focal or diffuse renal interstitial B-cell infiltration with tubular compression. Tubules and glomeruli as well as the glomerular basement membrane usually demonstrate normal morphology. Additional lesions such as acute tubular necrosis, glomerular lesions such as minimal change nephropathy, focal segmental glomerular sclerosis and light chain deposition disease may also be found.^4,8^ Kidney dysfunction due to LIK is rare and occurs in less than 1%.^4,7,9^ It is thought to result from compression of the tubular lumen, leading to intrarenal obstruction.

Light chain cast nephropathy is mostly seen in multiple myeloma^10^ but can be associated with Waldenström macroglobulinaemia^11^ and lymphomas of plasma cell origin^12,13^ as was seen in our patient. Excessive light chains produced by malignant plasma cells are filtered by the glomerulus and accumulate in the distal tubule, forming casts by binding the complementarity-determining portion of uromodulin.^10,13^ This results in tubular obstruction as well as direct tubular epithelial injury, ultimately resulting in AKI.

Kidney involvement by PBL in people living with HIV is rare with few case reports published.^13,14,15,16,17^ Most have reported urinary tract obstruction,^14,15,16,17^ with a single case report describing a LCCN.^13^ In a large case series of 112 HIV positive patients who were diagnosed with PBL, none of the patients had renal involvement,^18^ while in another smaller study that included 20 patients of whom 90% were diagnosed with Non-Hodgkin lymphoma (NHL) on kidney biopsy, none had the histological subtype of PBL.^19^ To our knowledge, our case is the first to describe both direct tumour infiltration and LCCN as a cause of AKI in an HIV patient with PBL.

The overall prognosis remains poor.^9^ In a study that included 135 patients with PBL, the median overall survival was only 32 months; however, HIV positive patients had a better survival on Kaplan–Meier analysis.^20^ More recently, in a large population-based study that included 248 patients, the reported 3-year and 5-year survival rates were 54% and 53%, respectively, and survival was not altered after adjusting for HIV status.^21^ As our patient had a very high tumour burden on bone marrow biopsy along with severe kidney dysfunction, the prognosis was deemed to be poor, and palliative care was instituted.

## Conclusion

To our knowledge, this case report represents the first description of dual pathology observed in a kidney biopsy as the underlying cause of AKI in an HIV-positive patient with PBL. While urinary tract obstruction is the most common mechanism by which PBL causes AKI, clinicians should maintain a high level of suspicion for multiple pathological processes in cases involving light chain producing PBL without evidence of urinary tract obstruction.

## Teaching points

1. Common mechanisms of AKI caused by lymphoma include urinary tract obstruction, tumour lysis syndrome, and consequences of nephrotoxic chemotherapy.
2. Kidney involvement by PBL in people living with HIV is rare.
3. Clinicians should maintain a high level of suspicion for multiple pathological processes in cases involving light chain producing PBL without evidence of urinary tract obstruction.
